# COVID-19 Pandemic in Mountainous Areas: Impact, Mitigation Strategies, and New Technologies in Search and Rescue Operations

**DOI:** 10.1089/ham.2020.0216

**Published:** 2021-09-13

**Authors:** Michiel J. van Veelen, Anna Voegele, Simon Rauch, Marc Kaufmann, Hermann Brugger, Giacomo Strapazzon

**Affiliations:** ^1^ Institute of Mountain Emergency Medicine, Eurac Research, Bolzano, Italy.; ^2^ Emergency Medical Services (COVID-1 Intermediate Care Unit), Bolzano Hospital, Bolzano, Italy.; ^3^ Department of Anesthesia and Intensive Care, “F. Tappeiner” Hospital, Merano, Italy.; ^4^ Emergency Medical Services 112, Health Care System Alto Adige, Bolzano, Italy.; ^5^ Department of Anesthesiology and Intensive Care Medicine, Medical University Innsbruck, Innsbruck, Austria.

**Keywords:** airborne infection, drone, mountain rescue, personal protective equipment, SAR

## Abstract

van Veelen, Michiel J., Anna Voegele, Simon Rauch, Marc Kaufmann, Hermann Brugger, and Giacomo Strapazzon. COVID-19 pandemic in mountainous areas: impact, mitigation strategies, and new technologies in search and rescue operations. *High Alt Med Biol*. 22:335–341, 2021.—Mitigating the spread of COVID-19, an airborne infection, can lead to delays in the prehospital response and impair the performance of search and rescue (SAR) services in mountainous and remote areas. We provide an overview of the developing epidemiological situation related to the COVID-19 pandemic in mountainous areas and review current protocols to determine their suitability for mountain rescue teams. We also discuss using novel technologies to reduce the adverse effects caused by COVID-19 mitigation strategies such as delays caused by donning personal protective equipment (PPE) and reduced rescuer performance due to impaired movement and ventilation. COVID-19 has spread even in mountainous and remote locations. Dedicated protocols for the use of PPE appropriate for SAR rescuers exerting physical effort in remote areas and using technologies such as drones, telemedicine, and localization and contact tracing applications could contribute to an effective and timely emergency response in mountainous and remote settings.

## Introduction

The COVID-19 pandemic is causing significant disruptions in health care systems worldwide, with over 100 million confirmed cases and over 2.5 million fatalities in the 1st year after being declared a pandemic (WHO, 2021). This significant burden of care influences all health care fields, including search and rescue (SAR) services. It is impossible to rule out infection in a victim before arrival or at the scene, necessitating time-consuming and possibly performance-impairing mitigation strategies to prevent viral transmission.

Mitigation strategies are required to keep the prehospital care level provided by SAR services effective and safe. We provide an overview of the developing epidemiological situation related to the COVID-19 pandemic in mountainous areas and review current protocols to determine their suitability for mountain rescue teams. We also discuss the perspectives of using novel technologies to reduce the possible negative effects caused by COVID-19 mitigation strategies.

## Impact of the Pandemic in Mountainous Areas

The COVID-19 pandemic has spread worldwide, including in mountainous areas, where it has an ongoing impact on the resident population, leisure-time activities, and accident management. A study published early in the pandemic reported that COVID-19 had a lower impact on inhabitants of high-altitude areas in Asia and South America than of lowlands (Arias-Reyes et al., 2020). The hypothesis that high altitude populations are protected from severe effects of COVID-19 through a superior response to hypoxemia has been proven to be wrong (Castagnetto et al., 2020; Intimayta-Escalante et al., 2020; Joyce et al., 2020; Pun et al., 2020).

**FIG. 1.**
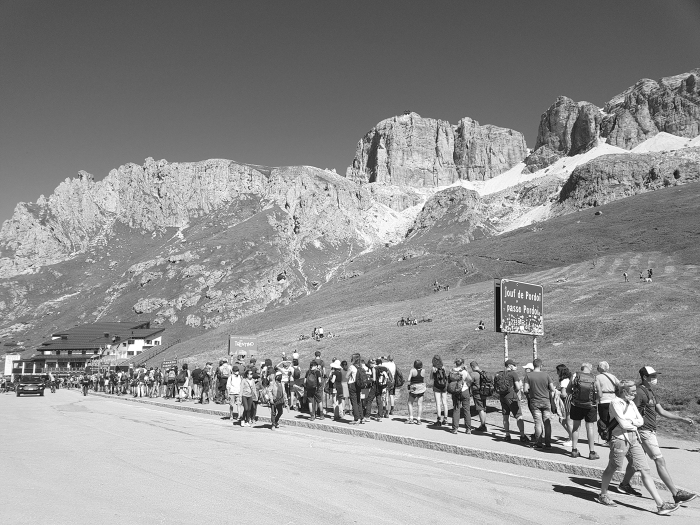
Long queues to access cable-car facilities in the Dolomites area, Italy, due to the unexpected afflux of tourists during summer 2020 after loosening of COVID-19 lockdown (courtesy of Marco Bassot).

High-altitude climbing destinations in South Asia have temporarily suspended climbing permits in an attempt to stop the spread of the COVID-19 infection since March 2020. Other mountainous regions such as the European Alps and the Rocky Mountains were also severely affected by the first wave of the COVID-19 pandemic. Before visitors were practicing social distancing and mitigation strategies in tourist infrastructures such as cable cars, mountain huts, and restaurants, outbreaks started in several ski resorts as early as February 2020. A retrospective survey in the Austrian town Ischgl showed the extent of a local outbreak; it revealed that 42% of the town's inhabitants tested positive for COVID-19 antibodies among 79% of the population tested (Knabl et al., 2020). Consequently, governments progressively shut down ski resorts in Europe and the United States in winter and spring of 2020 and the winter season of 2020/2021. For example, in the Dolomites, Italy, one of the world's largest ski-resort areas was shut down on March 9, 2020, due to the virus outbreak. The Italian government banned skiing to ensure that clinical services were fully available for COVID-19 patient care. Consequently, the number of tourist visits in the 2019–2020 skiing season decreased by 22% (Astatinfo, 2020). Trauma alerts for severe alpine trauma cases (i.e., with an injury severity score >16) recorded in the International Alpine Trauma Registry (Rauch et al., 2018) have shown a decrease of 75% from March to May 2020 compared to the same period in 2019 in this area (S. Rauch, pers. commun., January 2021). Governments granted residents more liberties in recreational sports after the first peak of the epidemic. The International Climbing and Mountaineering Federation (UIAA) has set up a COVID-19 Crisis Consultation task force. It regularly updates publicly accessible precautions on traveling to mountaineering destinations, staying in mountain huts, and informing on global trends and restrictions. A classification system associating mountain sports activities with infection risk levels was written by the UIAA Medical Commission, providing measures to reduce infection during mountain activities (UIAA, 2020). Following the initial strict countermeasures, mountainous areas and remote locations have become popular destinations again, and numbers of SAR activations increase during the summer. For example, Italy's Dolomites area reported an unexpected influx of tourists (Fig. 1) that pose a threat of COVID-19 transmission. This influx has led to an increase of 170% of SAR activations for severe alpine trauma cases between June and July compared to the same period in 2019, even with a reduced number of international visitors (S. Rauch, pers. commun., January 2021). Similarly, Yellowstone National Park, WY, hosted 955,645 recreation visits in July 2020. There was a 2% increase compared to July 2019, even with limited resort availability, no visitor centers open, and no sit-down dining permitted (National Park Service, 2020). These data suggest that even more people will perform their outdoor activities in mountainous areas, which will likely lead to a more significant burden on SAR services after lockdown periods due to the frequency of activations and the added challenge of utilizing mitigation strategies for airborne infection transmission.

## Current Mitigation Strategies and Possible Pitfalls

**FIG. 2.**
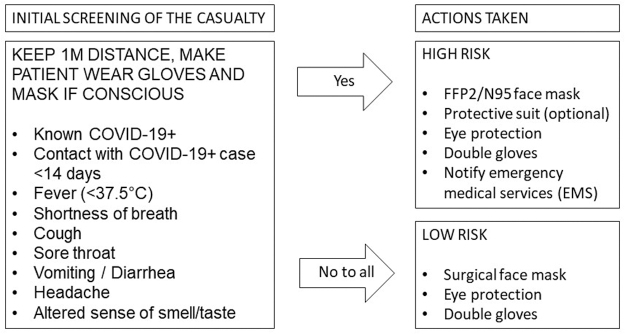
Initial approach algorithm of the Italian Corpo Nazionale Soccorso Alpino e Speleologico during the COVID-19 pandemic (modified from Masullo et al., 2020).

Emergency medical services (EMS) in Europe, Japan, and North America have developed internal guidelines on managing patient care during the COVID-19 pandemic and educating providers through statements, flowcharts, and videos (Centers for Disease Control and Prevention, 2020; Scuola Nazionale Direttori Operazioni di Soccorso, 2020). Currently, only the National Alpine and Speleological Rescue Corps of Italy has published a protocol specifically for the SAR setting (Masullo et al., 2020), as shown in Figure 2. In this approach, the outcome of a verbal screening assessment determines whether the provider should wear full personal protective equipment (PPE), including an FFP2/N95 face mask and optionally a protective suit or gown. This protocol utilizes a screening tool that could lead to a significant delay in approaching the patient. The reliability of the answers could be questionable under stressful and logistically challenging conditions. If PPE does not impair safety maneuvers, approaching every patient as initially high risk could save time and reduce infection risk, as the protocol advises wearing PPE continuously during the approach in the field or in vehicles and helicopters. The European Resuscitation Council also outlined explicit guidelines on practicing and training resuscitation, aimed to reduce the risk of exposure to aerosols (Nolan et al., 2020). The applicability of such a protocol to mountainous and remote environment has not been evaluated yet. A decision support tool for infection prevention in helicopter transport has also been published (Bredmose et al., 2020). Experiences with protocols specific to the SAR environment have been described, but there has been no structured evaluation of the effect on the quality of care delivered by rescuers (Kitchen, 2020).

**FIG. 3.**
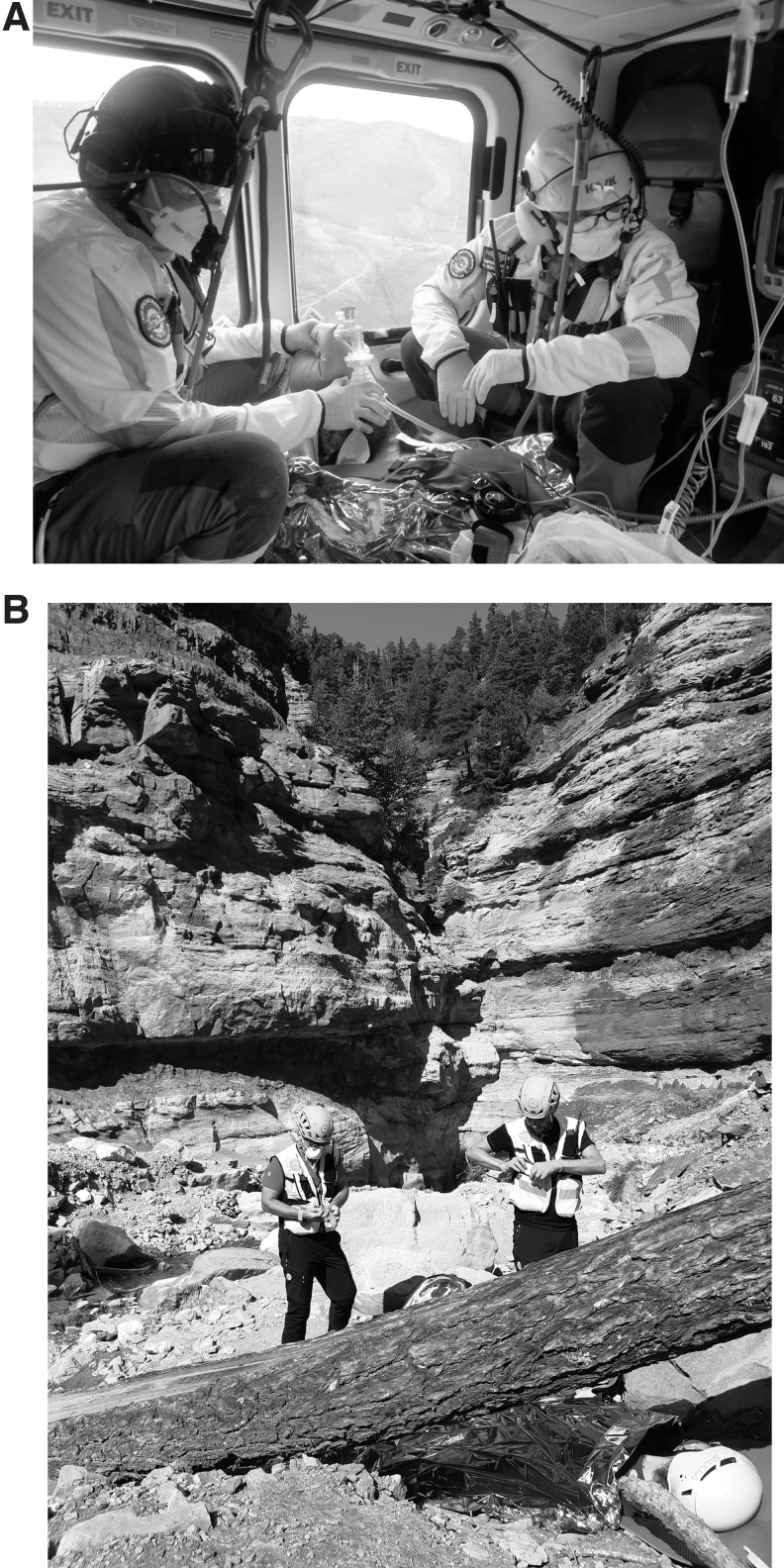
Use of PPE by SAR services. **(A)** Helicopter SAR personnel wearing PPE during transport. **(B)** SAR personnel wearing PPE during HEMS and ground SAR operations (courtesy of CNSAS, Gianluca Facchetti and Giacomo Strapazzon). CNSAS, Corpo Nazionale Soccorso Alpino e Speleologico; HEMS, helicopter emergency medical services; PPE, personal protective equipment; SAR, search and rescue.

A key decision point in protocols is whether members of SAR teams should continuously wear PPE during mountain and remote SAR operations (Fig. 3). Wearing PPE has a different impact on (sub)urban EMS providers than on SAR team members due to their different environmental exposure (Table 1). Wearing PPE can give a subjective sensation of impairment of breathing and limit dissipation of body heat (Morabito et al., 2020). Ground rescue operations are associated with high physical exertion, which leads to an increased breathing rate and respiratory minute volume (Callender et al., 2012). This effort increases the chances of infection and results in higher discomfort for providers wearing face masks. Wearing an FFP2/N95 equivalent face mask for over 10 minutes during regular exertion like walking causes subjective symptoms such as dyspnea, headache, fatigue, and impaired physical work capacity (Davis and Tsen, 2020). In healthy individuals comparing the effects of wearing no face mask versus a surgical mask versus an N95 mask performing a standard cycle ergometry ramp protocol, heart rate, respiratory rate, blood pressure, oxygen saturation, and time to exhaustion did not differ significantly. However, an increase of end-tidal carbon dioxide was measured while wearing N95 masks reaching a maximum of 8 mmHg at exhaustion (Epstein et al., 2021). Therefore, it seems safe and feasible for healthy individuals to perform short-term moderate-strenuous aerobic physical activity while wearing face masks and performing SAR activities such as cardiopulmonary resuscitation (CPR) (Rauch et al., 2021), ground transport, and winch operations. PPE could however increase the burden of increased body temperatures and heat-related illnesses, especially in a hot climate or in canyoning rescues (Strapazzon et al., 2018; Lipman et al., 2019). In addition, when wearing PPE, the face is partially covered and therefore communication among rescuers could be less effective (Hüfner et al., 2021), especially during acute management (Lim et al., 2020).

**Table 1.** Main Logistical and Environmental Factors Affecting the Impact of Wearing Personal Protective Equipment on Prehospital Emergency Medical Services Versus on Search and Rescue Crew Members

	EMS	SAR
Intensity of physical exercise	Moderate	Strenuous
Expected duration of application	<30 minutes	>30 minutes
Mobility required	Walking, patient transfers	Climbing, technical procedures
Environmental exposure (heat, cold, wind, precipitation, running water)	Rare	Common
Transportation	Ambulance predominantly	On foot, helicopter
Donning and doffing of PPE	Standardized setting	Improvised in the field

EMS, emergency medical services; PPE, personal protective equipment; SAR, search and rescue.

New PPE protocols against airborne infectious diseases should be compatible with technical equipment and specific environments such as snow and water in an avalanche, canyoning, or cave rescue (Schneider et al., 2016; Strapazzon and Brugger, 2018; Strapazzon et al., 2018). Disposable garments are fragile and do not stand up to the wear and tear of the prehospital wilderness environment. They become less effective if damaged and worsen working conditions during rescue operations, such as in high heat-stress situations or complex technical operations. Donning and doffing practices according to standardized operating procedures limit cross-contamination and require a rescuer dedicated to assisting (Masullo et al., 2020). Donning full PPE, including a gown, takes a minimum of 3 minutes in a hospital setting, measured as providers prepare for CPR during simulation training (Lim et al., 2020). When not utilizing these garments, disinfection strategies for conventional uniforms and technical equipment must be implemented to prevent cross-infection regarding not altering equipment integrity. Disinfection solutions used on contaminated surfaces could be sodium hypochlorite (0.1%–0.5%), 70% ethanol, or hydrogen peroxide solution (3%) by operators wearing adequate PPE (Masullo et al., 2020). Equipment not suitable for disinfection such as ropes and harnesses could be cleaned with lukewarm water with detergent solution or quarantined as advised by the manufacturer (Greatbatch et al., 2020).

SAR missions may require large rescue teams transported to the scene in confined spaces in helicopters or road vehicles. These gatherings make social distancing almost impossible and increase the risk of viral exposure. Multiple members work in close contact during rescue operations both for medical purposes and technical maneuvers. Such members could even be part of different organizations. Whether airborne PPE will be worn during the approach by all crew members should be defined by the team leader before the mission to guarantee safe working conditions depending on the specifics of the mission (e.g., long duration, difficult terrain). The number of members involved in patient transport and care should be minimized (Bredmose et al., 2020). SAR crews should consider packaging the patient to reduce infectivity before transport like wrapping the patient into a plastic sheet if continued access to the victim is not required. During transport in confined spaces, patients can be isolated instead of SAR and helicopter emergency medical services (HEMS) members using commercially available patient isolation transport units, requiring specific equipment and training (Albrecht et al., 2020). Nevertheless, this does not exclude the need for wearing PPE by HEMS and SAR members. Overall, resource allocation, impracticalities, and delays caused by wearing PPE and costs should be weighed against the staff's estimated infection risk, which would depend on the local prevalence and vaccination rate in a given period. Perspectives of regular testing to avoid sending infected rescuers to the spot and especially the globally increasing uptake of vaccines among rescuers could decrease the need and the level of PPE during the approach in open spaces among fully vaccinated. However, it is currently common practice to assess undifferentiated victims in appropriate PPE, regardless of the providers' vaccination status.

## Use of New Technologies in SAR

Applying PPE and screening for risk assessment delay rescue response and create new communication barriers between providers and victims that can interfere with medical interventions. The use of drones and other new technologies such as smartphone applications could partially remedy such issues.

Case series and experimental studies have shown the efficacy of drone assisted SAR missions in remote settings and extreme altitude and demonstrated a significant reduction in time to locate a victim, assist in triage, establish means of communication, and reduce workload requirement (Claesson et al., 2017; Karaca et al., 2018; McRae et al., 2019). Such capabilities could overcome delayed response times during the COVID-19 pandemic caused by application of PPE before the mission and allow a smaller rescue team to operate. Smaller teams require a smaller amount of PPE and limit close human interaction between rescuers during the approach and between rescuers and patients during the rescue, reducing the opportunity of viral spread. Especially in rugged terrain, using drones decreases the need for rescuers to cover the same area while wearing PPE, partially overcoming the challenge of PPE limiting breathing and dissipation of body heat. It is also feasible for drones to deliver medical equipment to the site of an emergency, such as an automated external defibrillator (AED) and first aid material (Mesar et al., 2018). This delivery shortens the treatment-free interval. Prompt initiation of bystander CPR before ambulance arrival leads to three times higher survival chances (Sasson et al., 2010). It would also be appropriate to deliver PPE, considering the low cost, weight, and volume and the added protection provided to bystanders and first responders. The correct application of PPE and initial treatment advice to a bystander such as the use of an AED can be explained through telemedical advice, which could be especially helpful in case of a bystander to overcome hesitation to assist out of fear of contracting COVID-19 (Sanfridsson et al., 2019; van Veelen et al., 2020). Limitations of using drones would be a restricted flight range due to battery capacity, a drone operator-dependent decreased maneuverability in unclear terrain, and limited diffusion and use among SAR operators.

GPS-based smartphone search applications such as Georesq or stand-alone GPS beacon devices such as SPOT could shorten activation time as a (high quality) phone signal is not needed to send out an alert, and speed location as coordinates is tracked in real-time and transmitted directly to the SAR services, optimizing human and logistical resources. Furthermore, the use of anonymized COVID-19 contact tracing applications installed on smartphones facilitates contact tracing after a mission by identifying the chain of contact of patients or providers tested positive for COVID-19. These applications utilize continuous Bluetooth signal emission and detection, which flags close contact with a positive COVID case, leading to an alert prompting the exposed person to get tested (Zastrow, 2020). This identification could offer maximum staff protection by promoting testing when indicated and avoiding unnecessary quarantines if exposure was not detected, preventing a shortage of available team members. Privacy concerns might impair user uptake, which is significant as digital contact tracing applications' effectiveness depends on a high population uptake and intervention timeliness (Braithwaite et al., 2020). Principal social rights such as privacy for providers and patients should always be safeguarded by voluntary participation and informing users of local data privacy regulations and conforming to them.

Education and training in new protocols utilizing PPE and novel technologies are crucial and provide essential guidance on further management. The main issues of working with PPE in acute care are safe and timely donning practices, likely requiring a buddy to prevent self-contamination, and safeguarding effective, closed-loop communication while wearing PPE (Lim et al., 2020; Masullo et al., 2020). Research questions open to assess the effect of implementing novel technologies in the field are evaluating actual shortening of time to locate victims utilizing drones, quantifying delays caused by initial screening and PPE donning procedures in the field, and delays in time to start treatment and initiation of evacuation in times of COVID-19. To evaluate the effectiveness of contact tracing applications and uptake among users in the field requires analysis of the number of users and the number of (false-positive or false-negative) alerts.

## Conclusion

Epidemiological data support the fact that COVID-19 occurs in mountainous areas. Current trends point toward rising numbers of visitors in these areas worldwide especially after lockdown periods, increasing the chances of transmission in case of a rescue. There is currently a scarcity of published protocols describing the use of PPE suitable for SAR rescuers to protect themselves from COVID-19. Therefore, we advocate for establishing concrete recommendations by an international body such as the International Commission for Mountain Emergency Medicine ICAR MedCom, in which input from experts worldwide, as well as from nonpublished protocols, can be incorporated. As the pandemic and its mitigation strategies evolve rapidly, guidelines will need to be updated regularly. The use of technologies such as drones, telemedicine, and mobile applications could contribute to an effective and timely emergency response in mountainous settings while limiting the risk of viral transmission. Compliance will depend on providers' successful education. Focused research is needed on the feasibility and outcome of implementing mitigation strategies and the use of novel technologies in the field to make recommendations for future uses.
